# Time-of-day effects on post-exercise phosphoproteomic profiling in mouse hippocampus

**DOI:** 10.3389/fcell.2026.1788597

**Published:** 2026-03-23

**Authors:** Ping Qian, Jinying Shen, Fangming Wang, Zhaoxu Lu, Dingding Cao, Luya Li, Feifei Ma, Sen Li, Zhuo Liu, Ting Zhang, Shan Wang, Jianxin Wu

**Affiliations:** 1 Beijing Municipal Key Laboratory of Child Development and Nutriomics, Capital Institute of Pediatrics, Beijing, China; 2 Department of Neurology, Capital Center for Children’s Health, Capital Medical University, Beijing, China; 3 Tongren Hospital, Capital Medical University, Beijing, China; 4 Respiratory Department, Capital Center for Children’s Health, Capital Medical University, Beijing, China

**Keywords:** acute exercise, circadian rhythm, hippocampus, phosphorylation, synapse

## Abstract

**Introduction:**

Exercise benefits cognition, and various aspects of exercise can impact physiological outcomes. Recently, there has been a growing interest in understanding the optimal timing of exercise to maximize its benefits. However, how timed exercise influences hippocampal function and signaling pathways remains largely unexplored. Therefore, our aim was to investigate the effects of exercise timing on hippocampal phosphoproteomic profiling.

**Methods:**

The phosphoproteome/phosphoproteome was conducted by affinity enrichment and liquid chromatography-tandem mass spectrometry on hippocampus of mice immediately after acute exercise or sham-exercise at the early rest phase (ZT3) or the early active phase (ZT15). To compare the differences in exercise-regulated phosphorylated sites and proteins between the two phases, bioinformatic analyses including functional enrichment analysis, motif analysis and kinase prediction were performed.

**Results:**

A bout of acute exercise induced significant changes in the phosphorylation status of 932 and 828 phosphosites during the rest and active phase, respectively. There were only 49 overlapped differentially regulated sites on 44 proteins. Functional enrichment analysis revealed that the breadth of signaling pathways modulated by exercise at ZT3 was considerably greater. The phosphorylation status of differentially phosphorylated proteins enriched in multiple pathways was vastly different after the timed exercise, so were the predicted upstream kinases. 29% differentially phosphorylated proteins were associated with synapse structure or function. Moreover, both timepoints converged on glutamate synapse-calcium signaling-LTP pathways via distinct molecular nodes. Daytime exercise increased the pCaMKII/CaMKII ratio (*p* = 0.04), while night-time exercise suppressed hippocampal GFAP (*p* = 0.02) and IBA1 expression (*p* = 0.01).

**Conclusion:**

Timed exercise elicited time-dependent phosphoproteomic features, and only a trivial number of phosphosites were regulated regardless of the time of exercise. Preliminary analyses suggest early daytime exercise may better support hippocampus-dependent learning, while early night-time exercise may be more beneficial for reducing neuroinflammation. This study advances exercise chronobiology and provides insights into circadian regulation of hippocampal exercise physiology, and involvement of epigenomic memory in this process would be interesting to be further studied.

## 1 Introduction

Exercise is recognized to help enhance or improve cognitive function and confer health benefits, having become a powerful auxiliary intervention for neurodegenerative diseases (Liu et al., 2026). In addition to the modality, intensity, frequency and duration, the timing of exercise is also suggested to be a critical modifier for physiological outputs (Gabriel and Zierath, 2019; Sasaki et al., 2014; Schroeder et al., 2012). And it remains an interesting question when exercise maximizes benefits. Recently, significant progress has been made in exercise chronobiology. It has been reported that humans often showed greater more increased strength, power and endurance in the afternoon/evening than in the morning (Bessot et al., 2006; Chtourou and Souissi, 2012; Fernandes et al., 2014). And exercise alone could cause similar phase delay as bright light (Youngstedt et al., 2016). On the level of molecular mechanisms, findings from rodents indicated that in skeletal muscle, exercise at the early active phase exerted a more robust metabolic response, including glycolysis, lipid oxidation, and BCAA breakdown (Sato et al., 2019); early daytime exercise triggered energy provisioning and tissue regeneration, while early night-time exercise activated stress-related and catabolic pathways (Maier et al., 2022). Several other tissues like bone (Xu et al., 2016; Yang and Meng, 2016) and adipose tissue (Christou et al., 2019; Paschos et al., 2012) have also been investigated for daily changes in physical performance. Similarly, exploring the role of time-of-day exercise on hippocampal functions has far-reaching significance in optimizing cognitive health and longevity. However, to our best knowledge, how timing exercise influences the function or signaling pathways in hippocampus remains virtually unexplored.

Converging evidence suggests that exercise plays essential roles in remodeling the structure and function of hippocampus via enhancing neurogenesis, accelerating new neuron maturation and promoting angiogenesis (Cooper et al., 2018; Marlatt et al., 2012; O'Callaghan et al., 2009). This process involves the activation of various signaling molecules, such as brain-derived neurotrophic factor (BDNF), tropomyosin receptor kinase B and cAMP response element-binding protein (CREB). Previous findings have indicated that the expression of proteins in hippocampus is relatively stable under both physiological and pathological conditions (Li et al., 2020; Qian et al., 2022a; Qian et al., 2022b). In contrast, protein post-translational modifications (PTMs) provide a dynamic and efficient molecular mechanism for integrating environmental and cellular information (Humphrey, James and Mann, 2015). Protein phosphorylation, a common PTM, is vital in regulating hippocampal signaling pathways involved in neuronal migration, axon growth, and synapse formation, such as mitogen-activated protein kinase (Lee and Kim, 2017) and cell cycle protein-dependent kinase 5 (Im et al., 2022) related pathways. Studies have demonstrated that daily voluntary wheel running (Chen and Russo-Neustadt, 2005) upregulated BDNF expression and activated P13K/Akt pathway. Exercise also exerts neuroprotective effects against high-fat diet induced hippocampal neuroinflammation by inhibiting TLR4 and phosphorylation of its downstream proteins (Kang et al., 2016). However, it is important to note that physiological adaptations to environmental stimuli in hippocampus are complex biological phenomena, which are often mediated by integrated networks of molecules across multiple pathways. Therefore, by studying integrated phosphorylation molecular networks, we could gain a better understanding of exercise chronobiology in hippocampus.

In this study, we aimed to investigate the specific and common phosphoproteomic signatures in the mouse hippocampus following a single bout of acute exercise performed at two counterbalanced timepoints in a day: the early rest phase (ZT3) and the early active phase (ZT15). Our findings revealed that exercise at the early rest phase exerted a broader impact on the biological functions. They both significantly affected pathways relevant to synaptic function, but with distinct phosphorylation sites or states. Preliminary analyses suggest a propensity that exercise at ZT3 is better for the hippocampal LTP, while exercise at ZT15 may be more beneficial in reducing neuroinflammation. These results highlight the characteristic changes in circadian rhythm that influence the exercise physiology in hippocampus, providing valuable insights into the molecular mechanisms underlying the effects of exercise timing on hippocampal function. Furthermore, investigating the potential involvement of epigenomic modifications (Kumar and Mohapatra, 2024) would prove insightful for advancing our understanding of exercise chronobiology and elucidating the molecular basis of circadian regulation in this context.

## 2 Materials and methods

### 2.1 Experimental animals

Male C57BL/6J mice (8–9 weeks old) were purchased from Charles River Laboratory and reared in the animal facilities of the Capital Institute of Pediatrics. Mice were fed *ad libitum* in temperature- and humidity-controlled condition (22 °C ± 2 °C) with a strict 12/12 h light/dark cycle (light on at 6:00 a.m.; zeitgeber time 0, ZT0). At 9–10 weeks of age, mice were randomly assigned into distinct groups according to receiving acute exercise (AE) or sham-exercise (SE, sedentary) treatment at ZT3 or ZT15: ZT3 AE, ZT3 SE, ZT15AE and ZT15SE (Sato, et al., 2019). AE mice were subjected to a single-bout of acute exercise for 1 h at ZT3 or ZT15 on a motorized treadmill. SE mice were placed on a stationary treadmill at ZT3 or ZT15 for 1 h.

**FIGURE 1:**
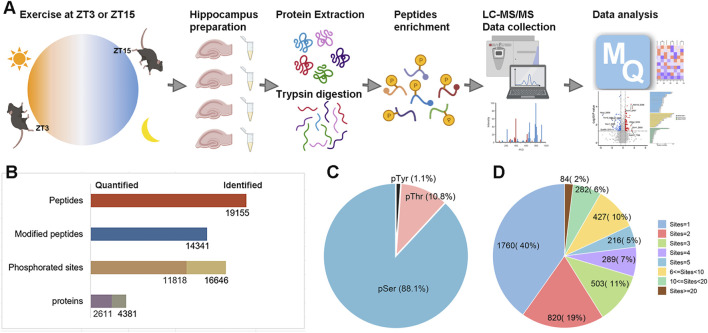
Overview of phosphoproteomic profiling in mouse hippocampus after timed exercise. **(A)** Experimental workflow for LC-MS/MS to identify hippocampal phosphoproteome after acute exercise at the early rest phase (ZT3) and the early active phase (ZT15). **(B)** Overall statistics for the hippocampal phosphoproteome. **(C)** Distribution of phosphorylation on the amino acid residues for all identified phosphosites. **(D)** Occupancy of the phosphorylation sites on per protein.

Immediately after the exercise, mice were euthanized by cervical dislocation (n = 10, Figure 1A). Each group of SE mice were sacrificed at a similar ZT period (time delay ≤30 min). Tail blood glucose was measured prior to sacrifice. Bilateral hippocampi were rapidly dissected out on ice-cooled surface, snapped frozen in liquid nitrogen and stored at −80 °C for subsequent use.

### 2.2 Acute exercise protocol

The acute exercise model was selected because PTMs are rapid, transient signaling events, and even a single 20-min bout of moderately intense exercise has been shown to induce transient modulation of cortical neural activity. The acute exercise protocol was adopted as described by Sato et al. (2019). Briefly, AE mice ran on a motorized treadmill with 5° incline, and underwent a 4-day’ acclimatization period, running for 15 min per day at gradually increasing speeds: starting at 6 m/min and increasing by 2 m/min every 2–3 min up to 12 m/min on Day 1, 14 m/min on Day 2, and 16 m/min on Day 3; this was followed by 1 day of rest. After this, the mice were subjected to a single bout of treadmill running at 16 m/min (80%–85% VO_2_max, (Fernando et al., 1993), for 1 h. Electrical stimuli of 0.6 mA were set to encourage mice to keep running at the back of each panel. During the training period of AE mice, the corresponding SE mice (ZT3 SE and ZT15 SE) were placed on a stationary treadmill with the same electrical stimuli.

### 2.3 Phosphoproteomic and proteomic sample preparation

Left hippocampi from every two mice were mixed as one sample and three replicates were adopted in each group. Process of peptide preparation was essentially similar as detailed previously with minor modifications (Qian et al., 2022a). Briefly, tissue was grinded and homogenized in lysis buffer (8 M urea, V900119-500G Sigma-Aldrich; 1% Protease Inhibitor Cocktail, 539134-10 ML Merck Millipore; 1% phosphatase inhibitor, 539133-1SET Merck Millipore). After sonication for three times (in ice water for 3 min with the following parameters: 3s of sonication alternating with 5 s of pause, at a power of 220W), the lysate was centrifuged at 12,000 g for 10 min at 4 °C. The supernatant was collected and the protein concentration was quantified with a BCA kit (Beyotime, Shanghai, China).

Protein lysate was subsequently reduced with 5 mM dithiothreitol at 56 °C for 30 min and alkylated with 11 mM iodoacetamide by incubating for 15 min at room temperature in dark. Thereafter, the samples underwent the first digestion with trypsin at 1:50 trypsin-to-protein mass ratio overnight and another 4-h digestion with trypsin at 1:100.

5 ug of the tryptic peptide was separated for proteomic analysis and 1 mg was used for phosphoproteomic analysis. For phosphopeptide enrichment, peptide mixture was first incubated with IMAC microspheres suspension and vibration in loading buffer (50% acetonitrile/0.5% acetic acid, pH 2-3 with a ratio of 1 mg of peptides to 18 µL of 50% IMAC beads). The IMAC microspheres were sequentially washed with 50% acetonitrile/0.5% acetic acid and 30% acetonitrile/0.1% trifluoroacetic acid to remove the non-specifically adsorbed peptides. The phosphopeptides were further eluted with buffer containing 10% NH4OH and then vibration. Finally, the supernatant containing the enriched phosphopeptides was collected and lyophilized for liquid chromatography-tandem mass spectrometry (LC-MS/MS) Analysis.

### 2.4 LC-MS/MS analysis

Peptides were analyzed on a nanoElute UHPLC system (Bruker Daltonics) coupled with the timsTOF Pro (Bruker Daltonics) mass spectrometry (PTM Biolab, Hangzhou, China). The peptides were dissolved in solvent A (0.1% formic acid and 2% acetonitrile in water) and loaded onto reversed-phase analytical column (25-cm length, 100 μm i.d.). Peptides for phosphoproteome were progressively separated with solvent B of 2∼22% over 72 min, 22∼35% for 14 min and 35∼80% for 5 min, and then hold at 90% for the last 5 min. The flow rate was 450 nL/min. Then the peptides were subjected to capillary source (CaptiveSpray) followed by the timsTOF Pro (Bruker Daltonics) mass spectrometry. The gradient of solvent B for separating peptides of proteome were the same as described previously (Qian et al., 2022a). For both phosphoproteome and proteome, the electrospray voltage was applied with 2.0 kV. Precursors and fragments were analyzed with a MS spectra range of 100–1,700 m/z. The timsTOF Pro mass spectrometry was operated in parallel accumulation serial fragmentation mode. Precursors with charge states 0 to 5 were selected for fragmentation, and 10 MS/MS scans/cycle were acquired per cycle. The dynamic exclusion was set to 30 s.

The resulting MS/MS data were searched against the Mus_musculus_10090_SP_20220107.fasta (17097 entries) database with MaxQuant search engine (v.1.6.15.0) with default parameters and 1% false discovery rate (FDR) for modified sites, peptide and protein. Trypsin/P was specified as cleavage enzyme, and up to 2 missing cleavages were allowed. The mass tolerance for precursor ions was set as 20 ppm in first search and 20 ppm in main search, and the mass tolerance for fragment ions was set as 20 ppm. Carbamidomethylation on Cys was set as a fixed modification. Phosphorylation on Serine, Threonine and Tyrosine were set as variable modifications for phosphopeptides analysis.

### 2.5 Bioinformatic analysis

#### 2.5.1 Comparison with public phosphorylation data

Known phosphosites were downloaded from public databases PHOSIDA ([http://141.61.102.18/phosida/index.aspx], downloaded in 03/2023) and PhosphoSitePlus ([http://www.phosphosite.org/], downloaded in 03/2023). New phosphosites were filtered out by comparing the identified sites in our study with sites in the two public databases.

#### 2.5.2 Functional enrichment analysis

Normalization to protein levels was first performed to eliminate the influence of protein expression on modification abundance. The fold-change ratio and *p*-value for AE relative to SE groups at ZT3 or ZT15 were calculated for each phosphosite. Cutoff of fold-change >1.5 or <0.67 and *p*-value <0.05 were defined as differentially upregulated or downregulated sites, respectively. Proteins containing such differentially phosphorylated sites (DPSs) were named as differentially phosphorylated proteins (DPPs) and subjected to Gene Ontology (GO) annotations and Kyoto Encyclopedia of Genes and Genomes (KEGG) database to identify functional features and pathways.

#### 2.5.3 Protein-protein interaction network

For DPPs, interactions with relatively high confidence were constructed for protein-protein interaction (De Matteis et al., 2013) network using STRING database (version 11.0), and visualized in R package “networkD3”. Using the MCODE plug-in, the most highly interconnected clusters were identified.

#### 2.5.4 Motif analysis

DPSs between AE group and SE group at ZT3 or ZT15 were investigated for consensus sequences with Motif-X algorithm in R package “motifx”. A window width of 15 residues central to Ser or Thr was investigated to determine the neighboring conserved sequences, which could partially reflect the characteristics of upstream kinases. The minimum occurrence of the sequences was set to 25 and significance threshold was set to 0.0001.

#### 2.5.5 Prediction for upstream kinases

Potential upstream kinases for DPSs were also predicted using NetworKIN (https://networkin.info/) with default parameters. Activity of these predicted upstream kinases was further analyzed by Gene Set Enrichment Analysis (GSEA).

### 2.6 Western blotting

Total protein was extracted from the right hippocampal tissue (n = 3) and then separated by 12% SDS polyacrylamide gel electrophoresis. The blots were incubated with monoclonal antibodies against rabbit β-actin (1:1,000, CST, United States), CAMKII (1:1,000, CST, United States) and p-CAMKII (1:1,000, CST, United States) at 4 °C overnight, followed by incubation for 1 h with an anti-rabbit enzyme-labeled antibody (1:5,000, CST, United States) at room temperature. After washing for 3–4 times, the membranes were processed with SuperSignal West Pico chemiluminescence substrate (Thermo, United States) and quantified with Quantity One software on a densitometer (Bio-Rad, Universal HoodII, United States).

### 2.7 Immunofluorescence

Immunofluorescence was performed as described previously (Qian et al., 2022a). Mice from each group (n = 4) were perfused transcardially with 50 mL of ice-cold saline and 50 mL of 4% paraformaldehyde successively. The removed brains were incubated in 4% paraformaldehyde overnight. Brain tissues were sectioned into 4 µm-thick slices. After dehydrated, the sections were treated with citrate buffer (pH 6.0), microwave and blocked with 10% goat serum. Subsequently, the sections were incubated with a primary antibody overnight at 4 °C [1:200 anti-ionized calcium binding adapter molecule 1 (IBA1) antibody, catalog number 17198S, CST; 1:200 mouse anti-glial fibrillary acidic protein (GFAP) antibody, catalog number 80788, CST], and then with a secondary antibody (catalog number GB21301, Servicebio) at room temperature for 1 h. After the last rinsing, the sections were stained with DAPI. Images were captured. Immunostained-positive GFAP and IBA1 cells were visually counted by two researchers blind to the intervention groups.

### 2.8 Statistical analysis

Values of blood glucose were expressed as mean ± standard deviation (SD) and statistical significance was assessed by two-way ANOVA analysis. Results of Western blotting and immunofluorescent staining were also shown as mean ± SD and statistical significance was performed by unpaired Student’s t-test. MS/MS data was firstly applied with log_2_ transformation of the relative quantitative value of proteins or phosphosites to make data closely follow normal distribution, then compared by unpaired Student’s t-test. The functional enrichment analysis for GO and KEGG was performed by Fisher’s exact test. *P* value <0.05 was considered statistically significant.

## 3 Results

### 3.1 Global phosphoproteomic profiling in mouse hippocampus

To investigate the specific impact of timed exercise on molecular pathways in hippocampus, mice were assigned into four groups: ZT3 AE, ZT3 SE, ZT15 AE and ZT15 SE. We observed that the basal blood glucose at ZT15 was lower than that at ZT3, and a significant decrease was only observed after ZT15 exercise (Supplementary Figure S1A), which might indicate exercise as a potential zeitgeber. For both phosphoproteome and proteome analyses, principal component analysis (PCA) displayed tight clustering within each group and clear segregation between groups (Supplementary Figures S1B,S1C), suggesting that the timed exercise imposed unique proteomic/phosphoproteomic signatures on hippocampus.

Across all samples, phosphoproteomic profiling identified a total of 16,646 unique phosphosites on 4,381 proteins (Figure 1B). Among these phosphosites, 14,660 were pSer (88.1%), 1,798 were pThr (10.8%), and 188 were pTyr (1.1%) sites (Figure 1C). Nearly 60% of the identified proteins harbored at least two phosphosites, and 18% contained five or more phosphosites (Figure 1D). By comparing the identified phosphosites with two other public databases, PHOSIDA and PhosphoSitePlus, we found that up to 3,478 phosphosites had never been reported before (Supplementary Table S1). These results provide valuable insights into the extensive phosphorylation landscape and highlight the potential regulatory role of phosphorylation in mediating the effects of timed exercise on hippocampal molecular pathways.

Based on the fold-change cutoff and *p*-value mentioned in the methods, 932 phosphosites on 648 proteins were differentially regulated for ZT3 AE vs. ZT3 SE (Supplementary Table S2), and 828 phosphosites on 585 proteins were differentially regulated for ZT15 AE vs. ZT15 SE (Supplementary Table S3). The ratio of DPSs to the quantified phosphosites was 7.8% and 6.9% for exercise at the rest phase and the active phase, respectively, corresponding to 24.9% and 22.3% proteins (Supplementary Figure S2A). Moreover, the majority (79.5%) of the differentially regulated phosphopeptides accounted for less than a quarter of the total cumulative phosphopeptide intensity (Supplementary Figure S2B), indicating their regulatory rather than structural roles (Robles et al., 2017).

### 3.2 Exercise elicited time-dependent phosphoproteomic features in hippocampus

**FIGURE 2:**
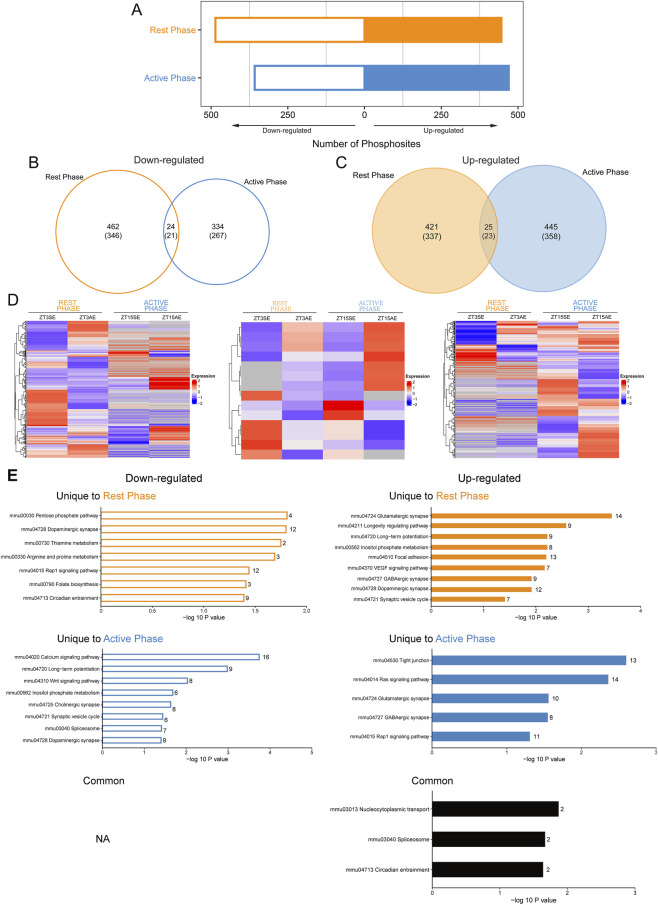
Distinct responses of hippocampal phosphoproteome to acute exercise at the early rest phase versus active phase. **(A)** The number of upregulated and downregulated phosphorylation sites in hippocampus immediately after exercise at the early rest phase (ZT3) and active phase (ZT15). **(B,C)** Venn diagrams showing the number of distinct and overlapped phosphorylated sites downregulated (left) or upregulated (right) by exercise between the two phases. The number of proteins containing such differentially phosphorylated sites are shown in the parentheses. **(D)** Heatmaps displaying the response of hippocampal phosphoproteome to exercise unique to the rest phase (left), overlapped for both phases (middle) and unique to the active phase (right). **(E)** KEGG pathway analysis of downregulated (left) and upregulated (right) phosphorylated proteins by exercise unique to rest phase or active phase, and overlapped for both phases. Numbers near each bar show the number of differentially phosphorylated proteins enriched in each pathway.

To probe the time-of-day specificity of hippocampal phosphoproteomic responses to acute exercise, we first compared the differences in phosphosites between AE and SE groups at each phase. Remarkably, exercise at the rest phase significantly upregulated 446 DPSs and downregulated 486 DPSs. Similarly, exercise at the active phase led to the significant upregulation of 470 DPSs and downregulation of 358 DPSs (Figure 2A). There was only a marginal overlap of DPSs between the two exercise phases (Figures 2B,C). GO analysis revealed that downregulated and upregulated DPPs at different exercise time were predicted to mainly function in synaptic components, whereas there existed notable differences in the most enriched molecular function and biological processes (Supplementary Figure S3). The upregulated DPPs at both phases were implicated in binding to cytoskeletal proteins and glutamate receptors, as well as in constituting the structural constituent of synapse. These proteins functioned in the development and morphogenesis of neuron projection and regulation of cation channel activity. Additionally, upregulated DPPs at rest phase were also involved in chemical synaptic transmission, regulation of neurogenesis and cation transmembrane transport, while those at the active phase were related to positive regulation of GTPase activity and maintenance of synapse structure. Downregulated DPPs at the rest phase were primarily involved in cellular differentiation and development, while those at active phase were related to protein depolymerization and calcium ion transport. These observations highlight distinct phosphoproteomic responses to exercise in mouse hippocampus that are unique to the time of day.

To gain further insights into the time-dependent phosphoproteomic signatures, we also focused on the pathways unique to each phase. KEGG analysis revealed that downregulated and upregulated DPPs unique to rest phase were highly enriched in 8 and 49 signaling pathways, respectively, while those unique to active phase were significantly enriched in 24 and 9 pathways, respectively (Supplementary Table S4). As displayed in Figures 2D,E, on one hand, proteins changed by exercise at the two counterbalanced phases and enriched in the same pathway showed the same phosphorylation trends, but had different phosphosites, such as glutamatergic synapse, GABAergic synapse and Ras signaling pathway; on the other hand, the overall phosphorylation status of proteins enriched in some pathways could be completely opposite, such as calcium signaling pathway, long-term potentiation (LTP) and insulin secretion. Collectively, the physiological effects of exercise at rest phase on hippocampus might be more extensive than those at the active phase, and timed exercise can affect the phosphorylation status on proteins even within the same pathways.

**FIGURE 3:**
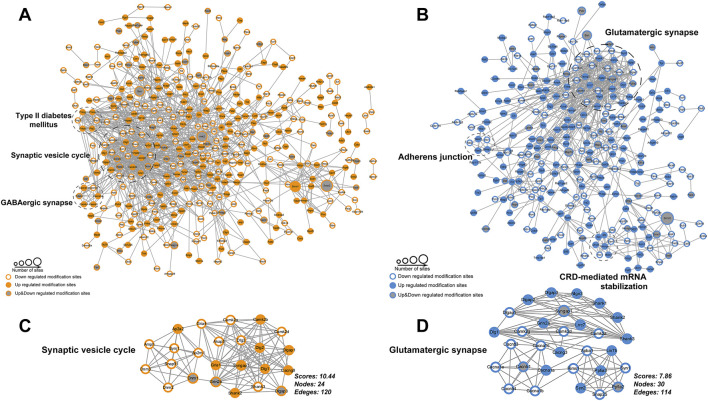
Protein-protein interaction network analysis. **(A,B)** Networks for differently phosphorylated proteins by exercise unique to rest phase **(A)** or active phase **(B)**; **(C,D)** Details for the most highly connected subnetwork corresponding to **(A)** and **(B)**, respectively.

PPI analysis (DPPs with confidence score>0.7) identified that the most highly connected clusters were also associated with synapse (Figures 3A,B). DPPs unique to the rest phase were clustered in synaptic vesicle cycle (Figure 3C) and GABAergic synapse, and those unique to the active phase were clustered in glutamatergic synapse (Figure 3D), adherent junction and CRD-mediated mRNA stabilization. These findings show the time-dependent phosphoproteomic signatures in the hippocampus in response to acute exercise, highlighting the importance of considering the timing of exercise in understanding its effects on molecular pathways and synaptic function in the hippocampus.

### 3.3 Analysis of overlapped DPSs

**FIGURE 4:**
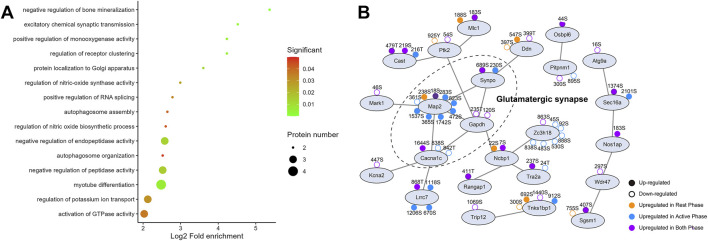
Functional annotation for the overlapped differentially phosphorylated proteins. **(A)** Bubble chart displaying the biological processes and **(B)** protein-protein interaction network analysis of these common proteins.

Although the vast majority of DPSs were regulated by exercise in the morning or evening, there were still a small number of DPSs regardless of the time of exercise. KEGG analysis showed that only proteins with 23 upregulated DPSs were predicted to be enriched in nucleocytoplasmic transport, spliceosome and circadian entrainment (Figures 2D,E). Further analysis of the biological function showed their involvement in negative regulation of bone mineralization, excitatory chemical synaptic transmission and positive regulation of monooxygenase activity (Figure 4A). And PPI network (confidence score>0.4) also centered on glutamatergic synapse (Figure 4B).

### 3.4 Motif analysis

Motif-X was used to identify overrepresented amino acid sequences surrounding DPSs, and a total of 122 motifs (103 pS and 19 pT) were identified (Supplementary Table S5). Apart from the overlapped sites, the sequence patterns were evenly distributed between DPSs specific to the rest phase and those specific to the active phase (Supplementary Figure S4). By comparison, among pSer (Supplementary Figure S4A), the recognition motifs unique to rest phase tended to be phosphorylated by basophilic kinases (188 basophilic verse 168 acidophilic), while motifs unique to active phase tended to be phosphorylated by acidophilic kinases (207 basophilic verse 220 acidophilic). Among pThr (Supplementary Figure S4B), both motifs unique to rest and active phases tended to be Proline-directed, as Proline located within ±3 positions of Thr sites. These results suggest that timed exercise likely regulated some distinct sets of kinases, which further influenced protein phosphorylation in hippocampus.

### 3.5 Prediction of potential kinases

**FIGURE 5:**
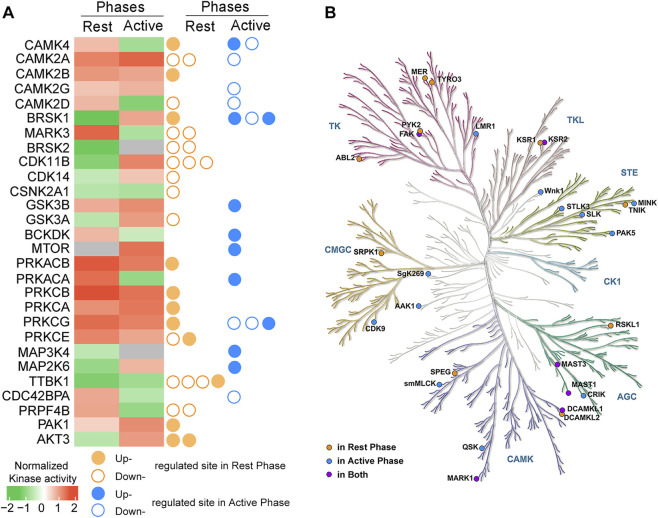
Analysis for potential kinases. **(A)** Activity and phosphorylation state of predicted kinases. Upstream kinases for differentially phosphorylated sites were predicted by NetworKIN and the activity of most predicted kinases were inferred by GSEA. The kinases themselves were significantly phosphorylated by exercise at either phase. Empty or filled circles indicate increased or decreased phosphorylation on each site. **(B)** The positions of 67 kinase-like phosphorylated proteins on the phylogenetic tree of the human kinome. Other than most predicted kinases by GSEA, timed exercise differentially regulated 67 kinase-like phosphorylated proteins which possessed protein kinase activity and/or functioned as part of a multi-subunit protein kinase complex, and these 67 proteins were matched on the phylogenetic tree of the human kinome.

NetworKIN was utilized to predict the potential kinases responsible for DPSs. A total of 47 upstream kinases were predicted, among which there were 41 kinases found to have at least two substrates (Supplementary Table S6). To substantiate the NetworKIN results, we employed GSEA to infer kinase activity based on known kinase-substrate relationships. Exercise at both timepoints were predicted to active several kinases, including CAMK2 (A/B/G), MARK3, PRKC (B/A/G/E), PRKACB, PKA1 and GSK3B, while inhibit CSNK2A1 and TTBK1. 13 kinases likely exerted opposite effects after exercise at rest phase compared to the active phase, such as AKT3, GSK3A, CAMK4 and CAMK2D (Figure 5A). Additionally, it is noteworthy that almost all the kinases themselves were modulated by phosphorylation at either phase (Figure 5A). And most kinases belonged to the CAMK2, PKA and PKC families, collectively acting upon 246 sites (Supplementary Figure S5).

To expand our prediction of potential kinases beyond the coverage of NetworKIN, we conducted the search within the list of DPPs for members that possessed protein kinase activity and/or functioned as part of a multi-subunit protein kinase complex (Potts et al., 2017). 95 kinase-like proteins were retrieved, including most of the predicted kinases by GSEA. For the remaining 67 kinases, their positions were plotted on the phylogenetic tree of the human kinome. The results showed that kinases regulated by exercise at rest phase belonged to kinome of CAMK, AGC and TK, while those regulated by exercise at active phase belonged to CAMK, AGC, CMGC and STG (Figure 5B). This comparative kinase analysis further supports the results obtained from motif prediction and highlights divergent regulation of kinase categories and activity between the two exercise models.

### 3.6 Timed exercise changed exiguous proteins

With the same filtering standard for DPSs, acute exercise at either phase induced exiguous changes in the hippocampal proteome (Supplementary Table S7). Specifically, 27 proteins were found to be differentially expressed after exercise at ZT3, with the upregulated proteins suggested to be involved in sodium ion/cation transport and RNA 3′terminal processing, while the downregulated were mainly associated with phosphatidylinositol synthesis or metabolism and the regulation of kinase activity. Exercise at ZT15 differentially regulated 15 proteins, with the upregulated proteins enriched in processes such as the negative regulation of DNA metabolism, assembly of ribonucleic acid protein complex and RNA splicing. The downregulated proteins in this case were mainly associated with monocarbonic acid synthesis and metabolism, as well as cellular responses to insulin stimulation (Supplementary Figure S6).

Considering that ZT3 and ZT15 are two stark converse timepoints in a day, it’s reasonable to investigate whether exercise influences the phosphorylation or expression of proteins related to circadian rhythm. Surprisingly, there was no significant enrichment of the circadian rhythm pathway in either omics data. Only a few proteins with increased phosphorylation were related to circadian rhythm, including PRKAB (107S), PRKAG (113S, 131S) and RBX1 (9T) at ZT3 (Supplementary Table S2), and PRKAG (65S, 161S) at ZT15 (Supplementary Table S3).

### 3.7 Timed exercise regulated phosphorylation on the molecular network related to synaptic plasticity

**FIGURE 6:**
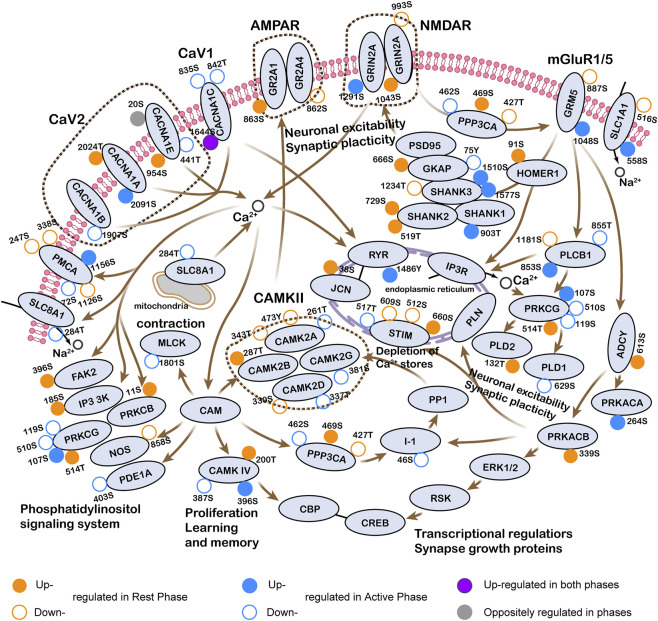
Integrated signaling pathway of glutamatergic synapse-Ca^2+^ signaling-long term potentiation. Particular phosphorylated sites on each protein regulated by exercise at either phase are indicated by the position and color around a circle. Empty or filled circles indicate increased or decreased phosphorylation on each site.

Synaptic plasticity is crucial for learning and memory, particularly through mechanisms of LTP and long-term depression. In our previous study, we demonstrated that chronic exercise enhanced hippocampus-dependent learning and memory, as well as adult neurogenesis (Qian et al., 2022a). In this study, we focused on exploring the potential impact of timed exercise on synaptic plasticity, specifically within the glutamatergic synapse-Ca^2+^ signaling-LTP pathway. This makes sense for several reasons. Firstly, we noted that a significant proportion of DPPs (29%, 358/1,233) were related with the structure or function of synapses. Secondly, DPPs were functionally enriched in various synaptic pathways following exercise at both rest and active phases, and some pathways contained proteins with opposite phosphorylation states, such as LTP and Ca^2+^ signaling pathway. Lastly, differentially expressed proteins upregulated by ZT3 or ZT15 exercise were found to be involved in ion transport or DNA/RNA processing. As shown in Figure 6, both ZT3 and ZT15 exercise might regulate the phosphorylation state and kinase activity of the CaV, AMPAR, NMDAR, mGluR, and CAMKII network, which could influence signal processing and structural remodeling in hippocampal synapses, thereby enhancing spatial memory encoding. Notably, CaMKII serves as one of the key kinases responsible for CREB phosphorylation; this event subsequently promotes the recruitment of transcriptional coactivators and the expression of numerous target genes. Further Western blotting analysis (Supplementary Figure S7) revealed that exercise at ZT3 reduced the total expression level of CaMKII while increased its phosphorylation ratio (pCaMKII/CaMKII) in the hippocampus (*P =* 0.04) while ZT15 exercise did not elicit similar changes. These findings suggest that early daytime exercise may be more effective than early night-time exercise in enhancing hippocampal synaptic plasticity and memory encoding for mice.

As extensive evidence has revealed anti-inflammatory role of physical activity, which is beneficial to synaptic function and neuroplasticity. Astrocytes and microglia are known to exert anti-inflammatory and neuroprotective functions via a variety of mechanisms. Therefore, we assessed GFAP-positive and IBA1-positive cells with immunofluorescent staining. Results (Supplementary Figure S8) showed that both the numbers of astrocytes (GFAP-positive cells, *p =* 0.02) and microglia (IBA1-positive cells, *p =* 0.01) were significantly lower in the running group of ZT15, while the number of IBA1-positive cells was significantly higher in the running group of ZT3 exercise (*p =* 0.01). These findings indicate that compared to exercise at ZT3, exercise at ZT15 might be more prone to play a vital role against inflammation.

## 4 Discussion

Physical activity has been widely acknowledged as a beneficial intervention for the prevention and treatment of nervous system diseases. Effects of exercise on the structure and function of hippocampus can vary depending on the exercise modes, intensity, frequency or duration. Driven by the inherent circadian rhythm, there is an interaction between exercise and the time of day (Ab Malik et al., 2020; Iwayama et al., 2015; Maier, et al., 2022; Rynders and Broussard, 2024; Sato, et al., 2019). Therefore, it is of great significance to explore the “optimal exercise time” to maximize exercise benefits. In this study, we utilized global proteomics and phosphoproteomics to analyze specific and common molecular signatures in the mouse hippocampus following acute exercise at different time of a day (at the early rest phase or the early active phase), providing novel insights into the biological responses to timed exercise.

In our study, we detected 16,646 phosphorylation sites, among which 3,478 sites were unreported previously. The considerable coverage of phosphorylation sites in hippocampus is comparable to that observed in the liver (Robles, et al., 2017), both of which are higher than that in skeletal muscle (Hoffman et al., 2015; Nelson et al., 2019) and myocardial tissue (Guo et al., 2017). Exercise at the rest phase significantly altered 7.8% (932/11,970) of phosphorylation sites, while exercise during the active phase affected 6.9% (828/12,007) of phosphorylation sites. These changes corresponded to 24.9% (648/2,607) and 22.3% (585/2,619) of proteins, respectively, with only 49 overlapping DPSs. Interestingly, the proportion of upregulated DPSs was equal to that of the downregulated. This is distinct from the changes observed after cerebral ischemia, which was dominant with downregulated phosphosites (Jiang et al., 2022). Functional enrichment analysis revealed that exercise at the rest phase influenced a broader range of signaling pathways compared to exercise at the active phase. The phosphorylation status of DPPs enriched in multiple pathways was vastly different after the timed exercise, so were some of the predicted upstream kinases. In contrast to the striking variation of phosphoproteome, only a small number of proteins were significantly regulated by exercise at ZT3 or ZT15 (27 and 15, respectively). This finding supports prior research showing that proteins maintain relative stability across a range of physiological and pathological states, including *C. neoformans* infection and epilepsy (Jiang, et al., 2022; Qian et al., 2022b). Together with our findings in chronic exercise remodeling lysine acetylome, we presume that the phosphorylation and acetylation on proteins are much more dynamic than proteins *per se*, emphasizing PTMs integrating information from cells and environment rapidly, efficiently and dynamically (Humphrey, et al., 2015; Shacter, Chock and Stadtman, 1984). In addition, though it has been reported that a single session of exercise is sufficient to shift the circadian phase in skeletal muscle (Kemler et al., 2020), our proteome and phosphoproteome did not detect any changes in core clock proteins. This is in line with another study investigating the circadian phosphoproteome in murine hippocampus (Chiang et al., 2017). Only several proteins with upregulated phosphorylation were found to be related to circadian rhythm pathway, including PRKAB (107S),PRKAG (113S, 131S) and RBX1 (9T) regulated by exercise at ZT3 (Supplementary Table S2), and PRKAG (65S, 161S) regulated by exercise at ZT15 (Supplementary Table S3). PRKAB/G is a subunit of AMP-activated protein kinase (AMPK), which plays a critical role in transmitting energy-dependent signals to the mammalian clock through driving the phosphorylation and destabilization of CRY and PER (Jordan and Lamia, 2013). The lack of significant changes in core clock proteins may be attributed to their high stability of protein expression and their low abundance.

Currently, a strong mutual interaction between circadian rhythm and exercise has been dominantly studied in skeletal muscle. For instance, exercise in the morning induced greater phosphorylation of M-band-associated proteins in human muscle, which might disrupt force transmission and potentially explain the lower knee extensor maximal voluntary isometric contraction force output in the morning (Ab Malik, et al., 2020). Maximal endurance performance was found higher in the early daytime for mice and timed exercise differentially altered the muscle phosphoproteome (Maier, et al., 2022) while Guo et al. (2025) found that mice underwent resistance training at ZT22 gained more muscle capacity and better metabolic fitness and metabolomics/lipidomics profiles under a high-fat diet. However, the influence of timed exercise on hippocampal adaptation has received less extensive investigation. Hwang et al. (2016) reported that improved memory and increased expression of synaptic plasticity-associated proteins by treadmill exercise were more prominent in mice exercising during the day or in the evening than that at dawn. Our screened phosphoproteome in mice’s hippocampus exercising at the early rest or active phase indicated that proteins enriched in the same pathway exhibited similar phosphorylation trends but with different phosphorylation sites or opposite phosphorylation status (Figures 2D,E; Supplementary Figure S6). They were predicted to share some common regulated kinases, while the activity of 13 kinases was affected in an inverse manner (Figure 5A). Based on these findings, we infer that, like skeletal muscle (Sato et al., 2019), protein phosphorylation in hippocampus also displays a time-of-day pattern upon exercise.

Notably, DPPs regulated by exercise at both the early rest and active phases were predicted to localize to the synaptic compartment, while exerted distinctly in the most enriched molecular function and biological process. Especially, DPPs upregulated by exercise at ZT3 were involved in chemical synaptic transmission and regulation of neurogenesis, while those at ZT15 were enriched in positive regulation of cation channel activity and maintenance of synapse structure. Consistent with previous work, synaptic proteins were conspicuously changed under several conditions of gut microbiota dysbiosis, inflammation, acute stress or aerobic exercise, suggesting that synapses are highly susceptible and adaptable to external stimuli. In rodents, the shift of dark-awake state leads to a rapid increase in dendritic spine density of CA1 pyramidal neurons, which is mediated by numerous kinase pathways (Ikeda et al., 2015). Protein kinase C (PRKCA, PRKCB, and PRKCG) and CAMK2B/CAMK2G have been found to be active during the sleep-wake transition (Bruning et al., 2019). Furthermore, coordination between the sleep-wake cycle and the circadian timing system is linked to structural plasticity within the hippocampus (Havekes et al., 2016). In our study, we also observed activation of protein kinase C, CAMK2B and CAMK2G by exercise during both phases (Figure 6). Therefore, it would be interesting to define the relationship between the phosphorylation of synaptic proteins regulated by timed exercise and the intrinsic circadian rhythm in hippocampus. Likewise, as ZT3 and ZT15 exercise caused extensive changes in glutamatergic and dopaminergic synaptic pathways, future research could explore the balance between excitation and inhibition of synapse through electrophysiological measurements.

LTP is a well-recognized cellular mechanism underlying learning and memory. It is reported that LTP peaked during the mice’s inactive phase, indicating that the hippocampus-dependent learning behavior in rodents was stronger in the day (Chaudhury and Colwell, 2002; Hoffmann and Balschun, 1992; Valentinuzzi et al., 2004). In this study, DPPs upregulated by exercise at ZT3 were enriched in the LTP pathway, while those enriched in the same pathway were downregulated DPPs by exercise at ZT15, especially the molecular network of CaV, AMPAR, NMDAR, mGluR, and CAMKII (Figure 6). Further laboratory Western blotting revealed higher pCAMKII/CAMKII only found in the running group of ZT3 (Supplementary Figure S7), which is consistent with the reported literature mentioned before. The underlying mechanisms for the difference might owe to daily variation of energy provision, NMDA receptor sensitivity, phosphatase activity and many other physiological and neurochemical processes such as hormone secretion, cellular communication, and even gene transcriptions. For example, insulin has been shown to increase the frequency of excitatory synapse current, raise the basal level of presynaptic terminal neurotransmitter release, and reduce the threshold of LTP induction (Labouebe et al., 2013). After exercising in the early daytime when energy substrates were sufficient, phosphorylation of the insulin secretion pathway is expected to be upregulated (Ashcroft and Hughes, 1990), potentially facilitating LTP amplitude.

Neuroinflammation is known to be associated with cognitive decline in aging and neurodegeneration, possibly by harming neuronal structures, changing neuroplasticity, and disrupting synaptic function. The brain’s resident immune cells, microglia and astrocytes, can change their morphology and functions to the proinflammatory state when activated (Di Benedetto et al., 2017). There is accumulating evidence that physical exercise can reduce inflammation, contributing to structural synaptic plasticity and cognitive improvement. Lee et al. (2015) reported that 6 weeks of exercise significantly attenuated the activation of microglia and astrocytes. In this study, we found that exercise at ZT15 inhibited the expression of GFAP and IBA1 in hippocampus (Supplementary Figure S8), indicating weaker inflammatory state compared with exercise at ZT3. This suggests that for individuals with chronic metabolic diseases or neurodegenerative disorders, engaging in physical activity in the early daytime may be more beneficial in reducing neuroinflammatory responses and improving cognitive function.

Despite the vast time-dependent regulation of phosphorylation sites, exercise changed a small number of protein phosphorylation independent of the time. These DPPs were mainly involved in negative regulation of bone mineralization, excitatory chemical synaptic transmission and positive regulation of monooxygenase activity. And the 23 upregulated DPPs were enriched in nucleocytoplasmic transport, spliceosome and circadian entrainment (Figure 2D). The precise mechanism underlying these effects is not yet clear. It is possible that both direct signal transduction (receptor driven kinase cascades) or indirect effects (cortisol levels and body temperature changes) could be involved. Considering inhibition of Ca^2+^ channel negatively regulates the neurotransmitter release (such as γ- Aminobutyric acid, dopamine and glutamic acid) and associated signal transduction, we speculate that, regardless of the time of exercise, shortly after acute exercise itself, negative regulation of bone mineralization could increase circulating Ca^2+^ concentration, which could promote excitatory chemical synapse transmission (Liu et al., 2018). Additionally, recent research has highlighted the relevance of abnormal spliceosome in conditions like obesity (Pihlajamaki et al., 2011; Vernia et al., 2016) and depression (Wang et al., 2020). Understanding the regulation of exercise on spliceosome function may therefore have implications for the prevention or treatment of these diseases.

Exercise is widely recognized for improving metabolic health by enhancing insulin sensitivity and glucose metabolism. Clinical and preclinical studies support time-dependent effects: diabetes patients show better 24-h blood glucose control with afternoon versus morning exercise (Savikj et al., 2019), while rodents exhibit increased glycogen/glucose consumption after nocturnal exercise (Maier et al., 2022; Sato et al., 2022), and exercise at ZT23 elicits more energy metabolism-related transcriptional changes in adipose tissue than ZT13 (Kutsenko et al., 2025). Consistent with these findings, our study demonstrated lower blood glucose at ZT15 than ZT3, with a further ZT15 exercise-induced decrease associated with downregulated phosphorylation of the hippocampal insulin secretion pathway (Supplementary Figure S6). This aligns with Pendergrast et al. (2023), who reported that only active-phase exercise activates adipose tissue lipolysis to synergistically reduce blood glucose, an effect driven by the circadian clock rather than feeding status. Collectively, these data indicate a systemic network regulating glucose metabolism via exercise. Notably, unlike skeletal muscle (enhanced glycolysis) or adipose tissue (activated lipolysis) during active-phase exercise (Sato et al., 2019; Pendergrass et al., 2023), the hippocampus—an essential cognitive organ—only indirectly contributes to glucose homeostasis through insulin signaling pathway phosphorylation. This distinction reflects the hippocampus’s core role in energy sensing and cognitive integration rather than direct metabolic substrate breakdown, supporting a tissue-specific division of labor in the time-dependent metabolic effects of exercise.

We fully acknowledge that long-term repeated exercise interventions elicit cumulative and adaptive physiological effects that that are fundamentally distinct from the acute exercise responses characterized in this study. In contrast to the immediate, transient phosphoproteomic signaling events captured in our acute exercise model, which reflect the primary, circadian-dependent hippocampal response to exercise timing, unconfounded by cumulative structural or functional remodeling-chronic training would drive sustained adaptive changes in the hippocampus. This included persistent remodeling of synaptic plasticity pathways, circadian entrainment of hippocampal molecular clocks, and coordinated systemic metabolic and neurohumoral interactions between the hippocampus and peripheral tissues. Consequently, our acute exercise findings should be interpreted not as the end-stage adaptive outcome of long-term exercise, but rather as the initial molecular blueprint underlying hippocampal responsiveness to exercise timing. Moreover, the phase-specific molecular responses identified (e.g., enhanced LTP-related phosphorylation at ZT3 and suppressed neuroinflammatory marker expression at ZT15) provide a critical mechanistic rationale for designing personalized timed chronic exercise interventions. Future studies will validate these acute phosphoproteomic findings in a long-term training paradigm to further bridge the gap between acute and chronic exercise effects and enhance the translational value of our work.

In conclusion, a comprehensive understanding of exercise benefits, including the exercise model, intensity, duration, and “when to exercise”, provides a far-reaching step forward in harnessing this lifestyle intervention to improve health (Neufer et al., 2015). With affinity enrichment and LC-MS/MS, this study compared the proteomic/phosphoproteomic changes induced by acute exercise at the early rest phase verse early active phase in the mouse hippocampus. The phosphoproteomic analysis showed time-of-day specific responses to exercise, involving various types of synapses, LTP, calcium signaling pathways, insulin secretion, etc. Only 49 DPPs overlapped between exercise at both phases. Upstream kinase prediction identified that exercise at different time activated some protein kinase C and Ca^2+^/calmodulin dependent kinase 2, but activity of half of these kinases was opposite. Contrary to DPPs, only 42 proteins were significantly altered by timed exercise. Exercise did not alter any circadian rhythm core proteins, but differentially phosphorylated AMPK subunits and RBX1. In addition, preliminary analyses suggest a propensity that exercise in the day is better for the hippocampus-dependent learning behavior, while exercise in the evening may be more beneficial in reducing neuroinflammation. Although much remains to be learned about the difference of the altered phosphorylation network after timed exercise, the present study enriches the emerging resources of exercise chronobiology. It provides a new perspective for mechanistic understanding of exercise’s regulation upon hippocampus, and lays a theoretical foundation to explore how exercise, as a zeitgeber, can prevent or rehabilitate neuropsychiatric diseases. Notably, further investigation into the potential involvement of epigenomic marks could offer an additional layer of mechanistic insight into exercise chronobiology and its beneficial effects.

## Data Availability

The datasets presented in this study can be found in online repositories. The names of the repository/repositories and accession number(s) can be found below: http://www.proteomexchange.org/,PXD043450.
