# Supplementary figures and images for: Time-of-day effects on post-exercise phosphoproteomic profiling in mouse hippocampus
Source: Front Cell Dev Biol. 2026 Mar 23;14:1788597. doi: 10.3389/fcell.2026.1788597 (PMC13050938; doi:10.3389/fcell.2026.1788597)

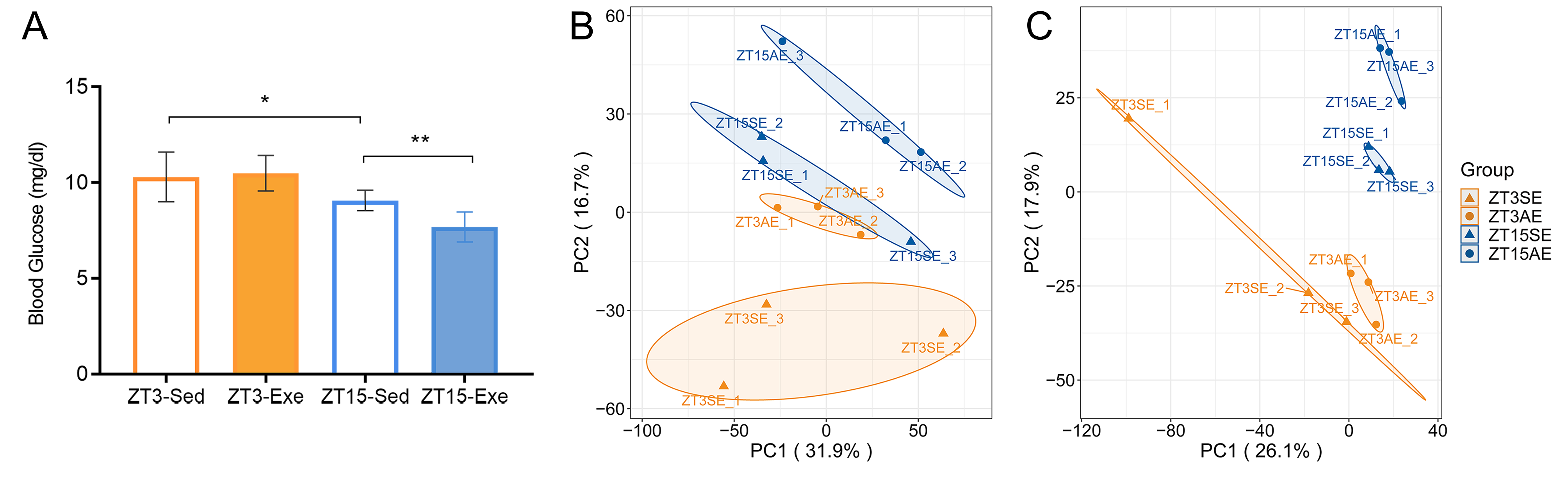

Supplement: Supplementary file 1 [file DataSheet1.zip › Supplementary/Fig.S1.tiff]

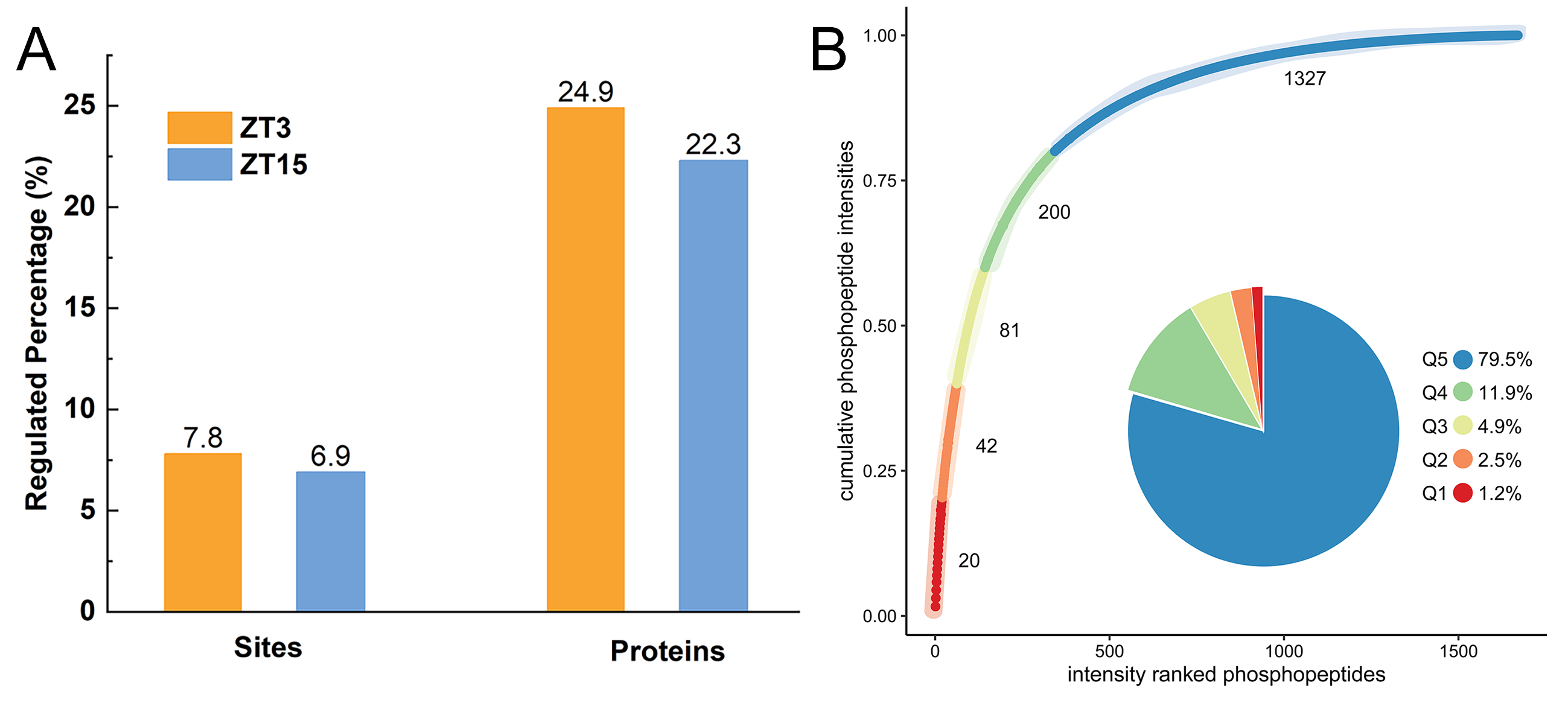

Supplement: Supplementary file 1 [file DataSheet1.zip › Supplementary/Fig.S2.tif]

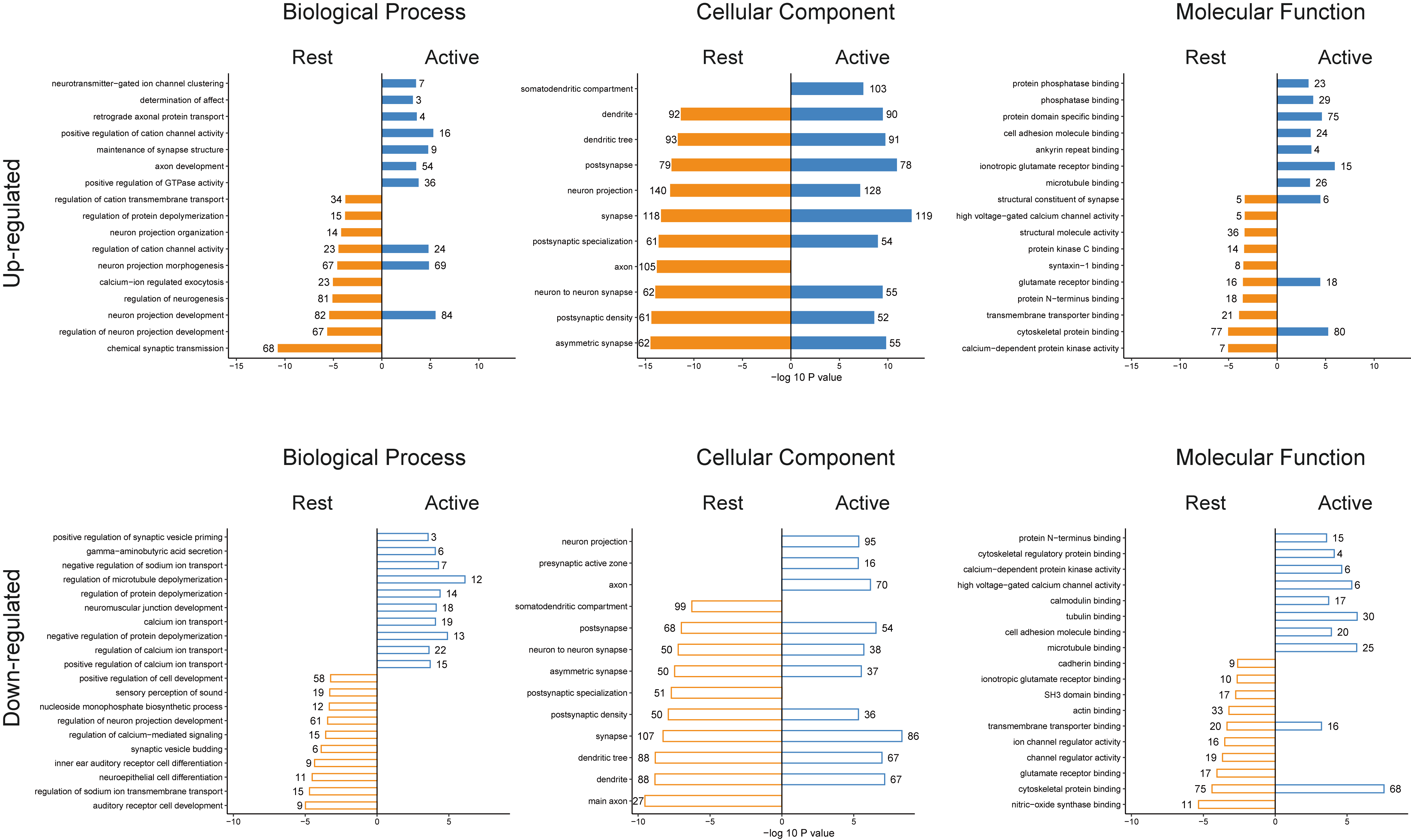

Supplement: Supplementary file 1 [file DataSheet1.zip › Supplementary/Fig.S3.png]

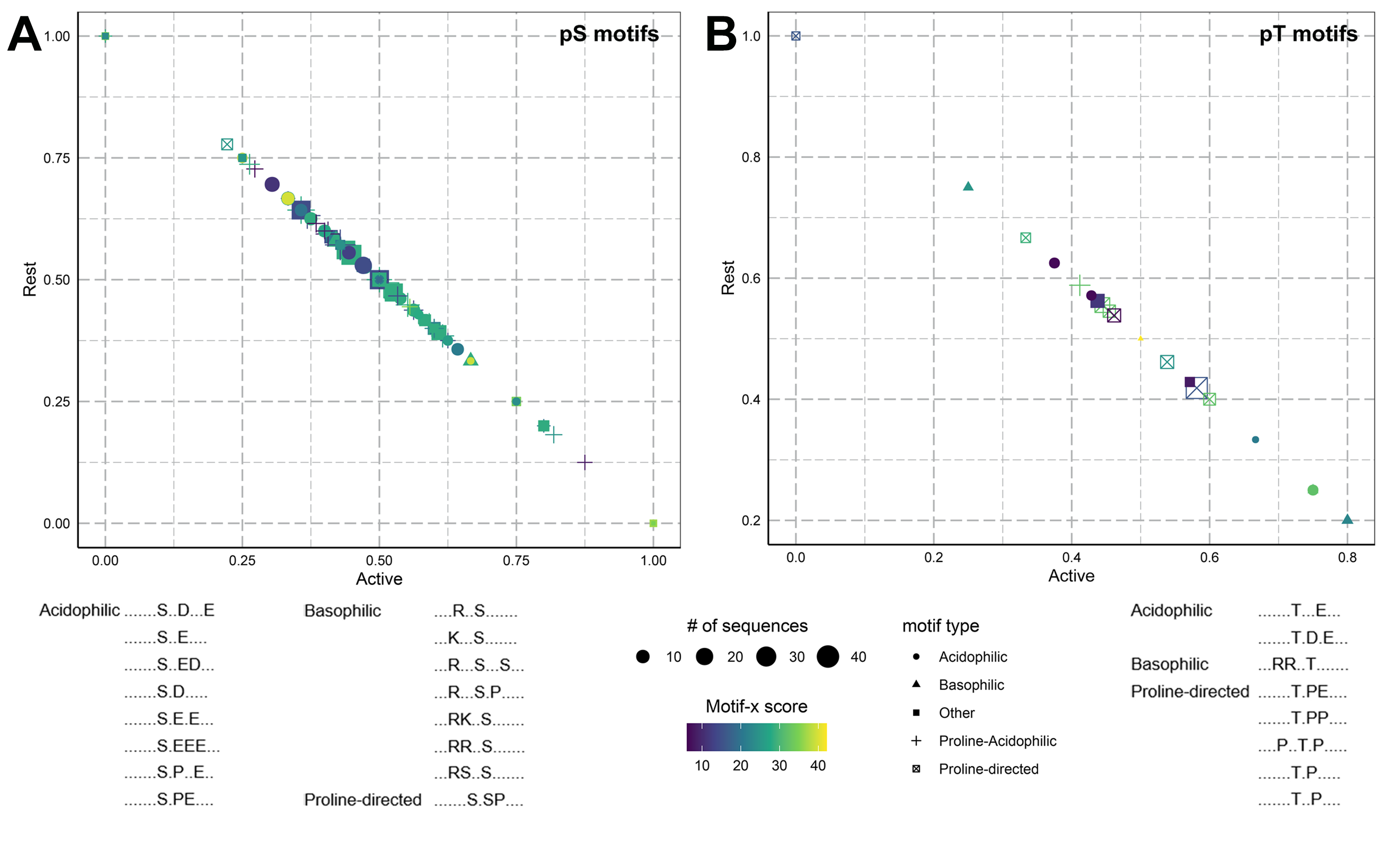

Supplement: Supplementary file 1 [file DataSheet1.zip › Supplementary/Fig.S4.tif]

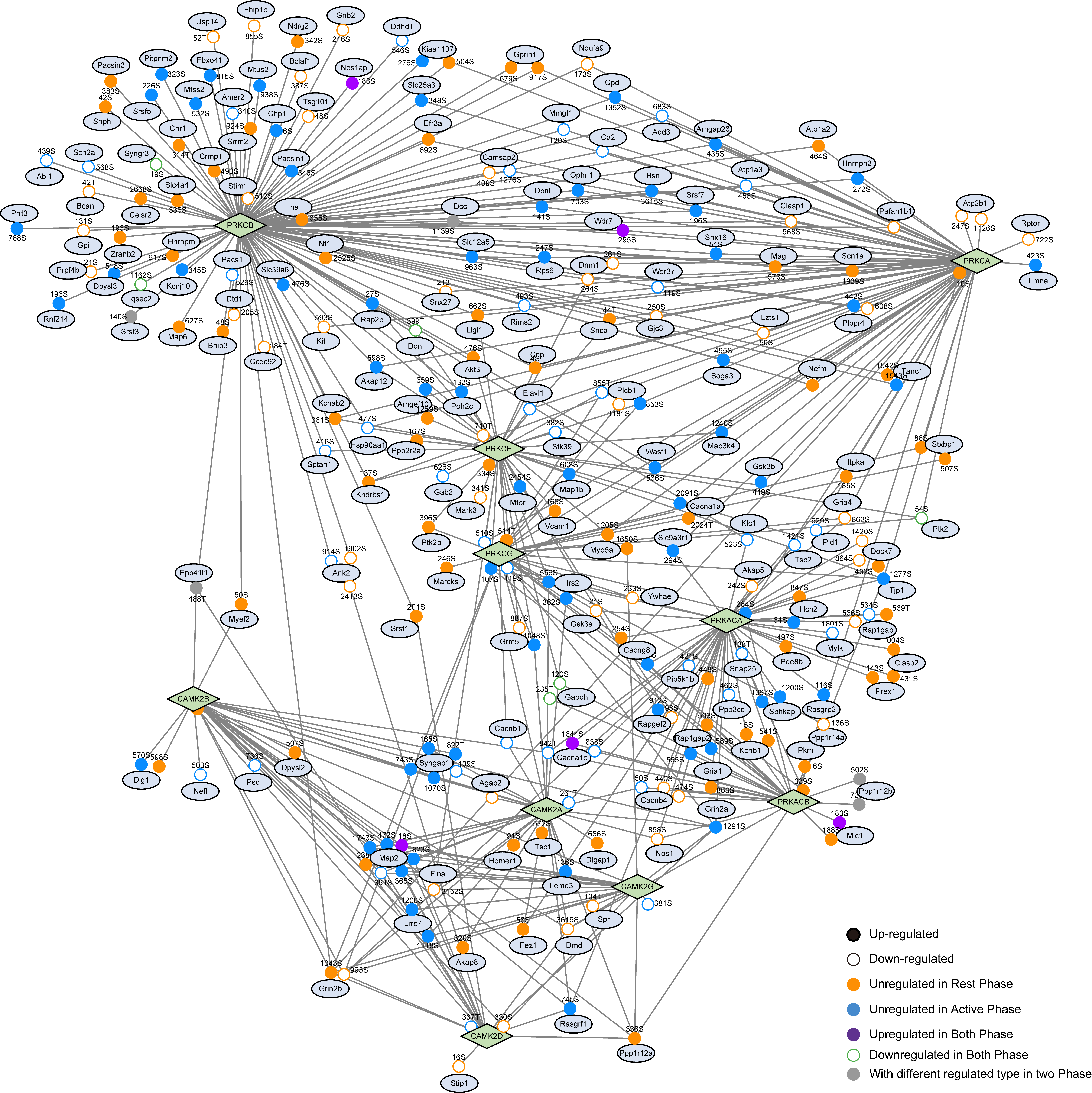

Supplement: Supplementary file 1 [file DataSheet1.zip › Supplementary/Fig.S5.png]

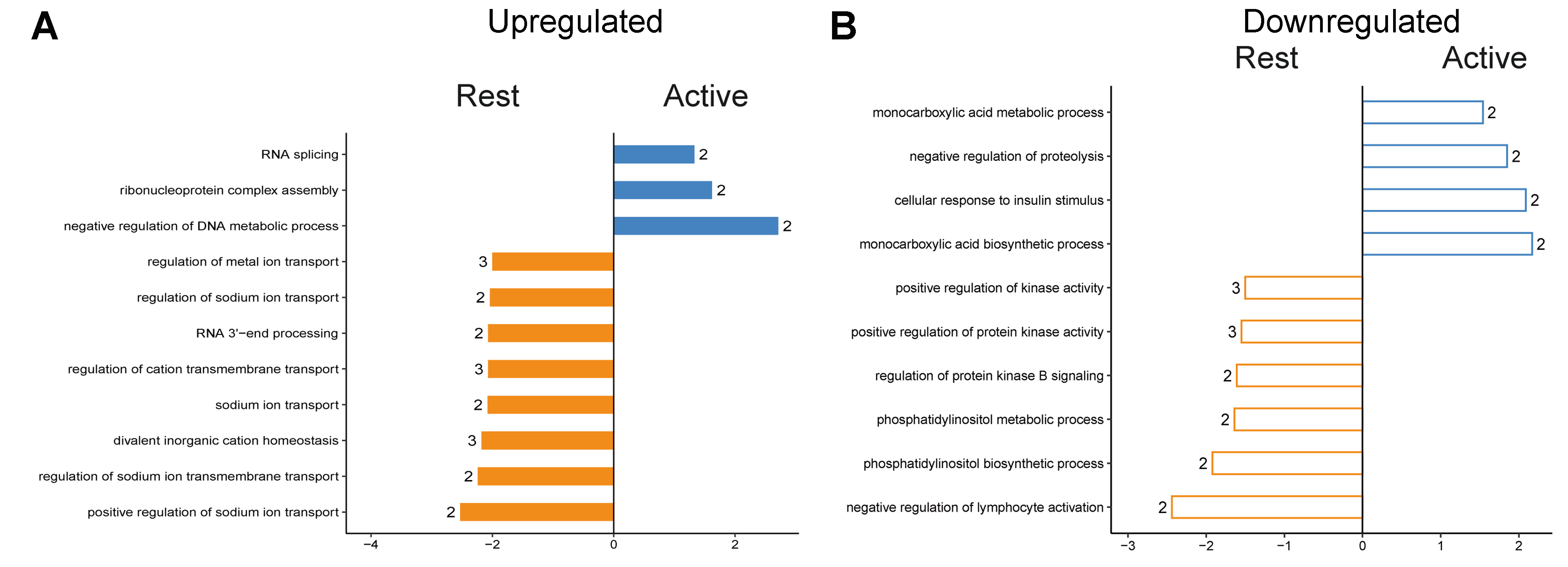

Supplement: Supplementary file 1 [file DataSheet1.zip › Supplementary/Fig.S6.tif]

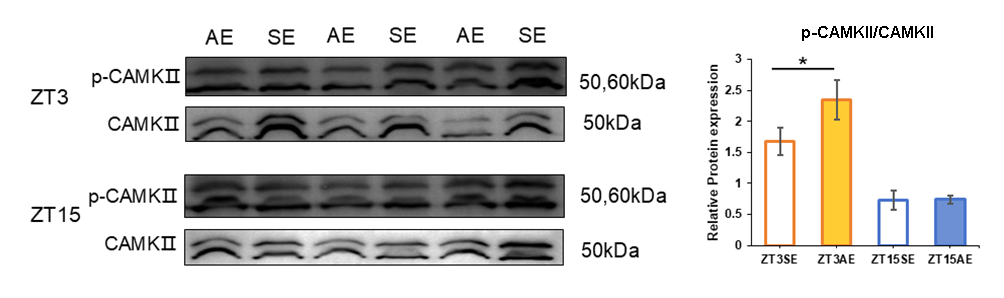

Supplement: Supplementary file 1 [file DataSheet1.zip › Supplementary/Fig.S7.tiff]

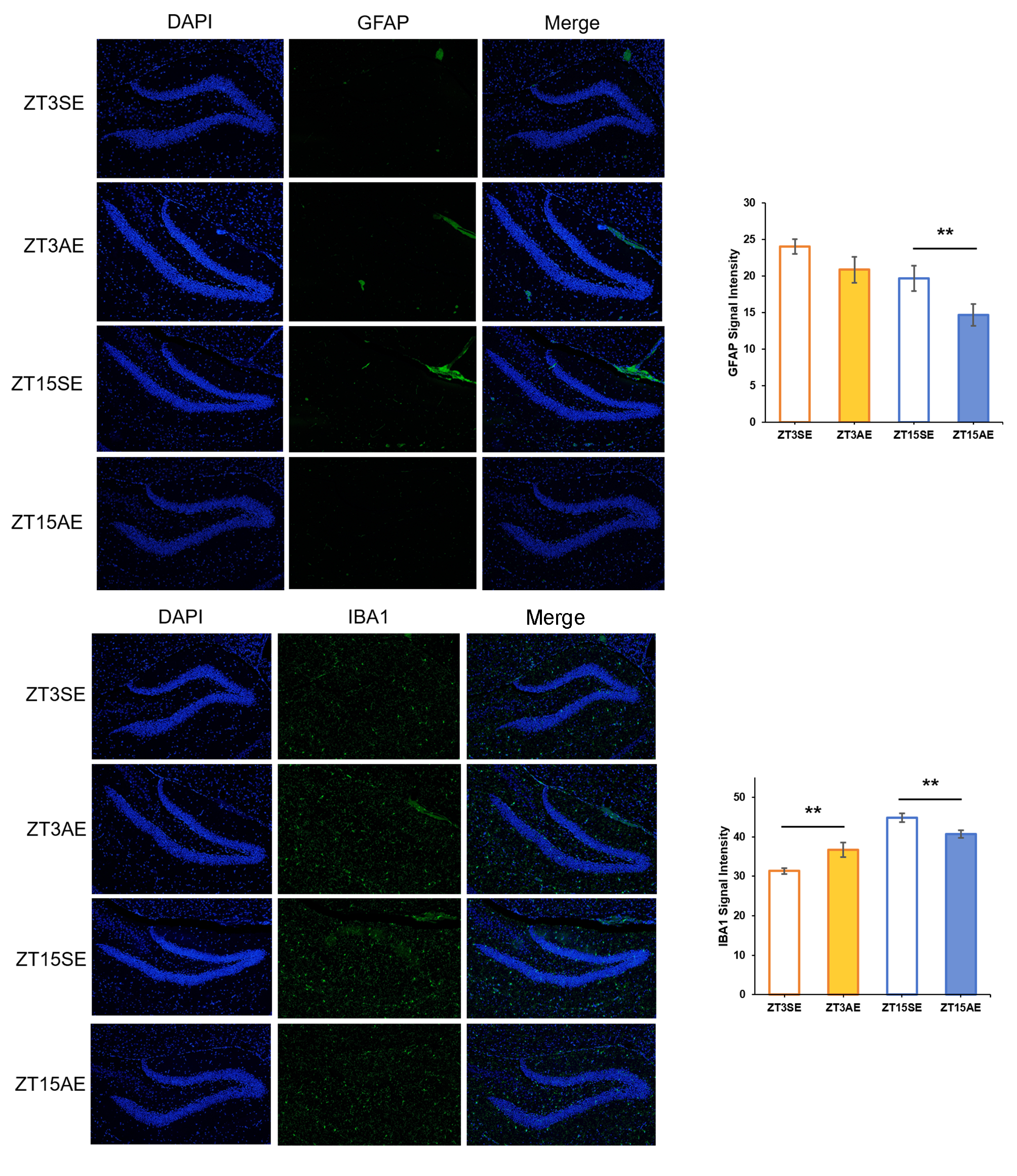

Supplement: Supplementary file 1 [file DataSheet1.zip › Supplementary/Fig.S8.tif]
